# Determining Equine Influenza Virus Vaccine Efficacy—The Specific Contribution of Strain Versus Other Vaccine Attributes

**DOI:** 10.3390/vaccines8030501

**Published:** 2020-09-03

**Authors:** Sylvia Reemers, Denny Sonnemans, Linda Horspool, Sander van Bommel, Qi Cao, Saskia van de Zande

**Affiliations:** MSD Animal Health, Wim de Körverstraat 35, 5831 AN Boxmeer, The Netherlands; sylvia.reemers@merck.com (S.R.); denny.sonnemans@merck.com (D.S.); sander.vanbommel@merck.com (S.v.B.); qi.cao2@merck.com (Q.C.); saskia.vandezande@merck.com (S.v.d.Z.)

**Keywords:** equine influenza virus, vaccine, strain, sublineage, infection, viral shedding, protection

## Abstract

Vaccination is an effective tool to limit equine influenza virus (EIV H3N8) infection, a contagious respiratory disease with potentially huge economic impact. The study assessed the effects of antigenic change on vaccine efficacy and the need for strain update. Horses were vaccinated (V1 and V2) with an ISCOMatrix-adjuvanted, whole inactivated virus vaccine (Equilis Prequenza, group 2, FC1 and European strains) or a carbomer-adjuvanted, modified vector vaccine (ProteqFlu, group 3, FC1 and FC2 HA genes). Serology (SRH, HI, VN), clinical signs and viral shedding were assessed in comparison to unvaccinated control horses. The hypothesis was that group 2 (no FC2 vaccine strain) would be less well protected than group 3 following experimental infection with a recent FC2 field strain (A/equi-2/Wexford/14) 4.5 months after vaccination. All vaccinated horses had antibody titres to FC1 and FC2. After challenge, serology increased more markedly in group 3 than in group 2. Vaccinated horses had significantly lower total clinical scores and viral shedding. Unexpectedly, viral RNA shedding was significantly lower in group 2 than in group 3. Vaccination induced protective antibody titres to FC1 and FC2 and reduced clinical signs and viral shedding. The two tested vaccines provided equivalent protection against a recent FC2 EIV field strain.

## 1. Introduction

Equine influenza (EI), caused by equine influenza virus (EIV), is highly contagious and is one of the major viral respiratory diseases in horses [1]. Equine influenza outbreaks occur worldwide (except in Iceland, New Zealand, and Australia) with high morbidity and potentially huge economic consequences [2,3]. EIV infection produces a considerable spectrum of clinical disease, with characteristic clinical signs seen in naïve horses and less severe disease generally seen in vaccinated horses, with subclinical or mild and transient clinical signs. There is evidence that infection with different EIV strains (genotypes) results in different clinical outcomes (disease-causing phenotypes). Certain strains appear more likely to produce primary viral pneumonia and others are more likely to be followed by secondary bacterial infection, while the remainder produce less severe disease.

The most effective method of limiting EIV infection and outbreaks is vaccination [4]. Several EIV vaccines are available commercially and these can be divided into three groups based on vaccine type: 1―adjuvanted, whole or subunit, inactivated virus vaccines; 2―adjuvanted, modified viral vector vaccine; 3―live attenuated virus vaccine [4]. An adjuvant is included to stimulate the host immune response to the target antigen(s) in the whole inactivated or subunit virus vaccines and modified viral vector vaccine. The adjuvanted vaccines are administered by deep intramuscular injection and the live attenuated vaccine by intranasal application.

Population (herd) vaccination coverage is needed to control EI. However, many horse owners are unaware that vaccination does not protect an individual horse from infection and disease but helps to reduce the prevalence and severity of disease. Moreover, a small percentage of the population fails to mount and/or maintain an adequate immune response after appropriate immunisation and, therefore, remains susceptible to EI despite having been vaccinated [5,6,7]. Although the reasons for this remain unclear, age appears to be a significant factor [8]. There is also likely to be a strong host effect, with genotype and previous immune experience also playing roles [9,10]. The timing of vaccination is important. A primary vaccination course usually consists of two vaccinations (V1 and V2) followed by an initial revaccination (V3) 5–6 months after the primary vaccination course. In fact, EIV-specific antibody titres can decline significantly between V2 and V3, meaning that some horses may be protected insufficiently for several weeks [11,12]. This so-called immunity gap poses a risk to all EI vaccines [13]. Horses infected during the immunity gap may show little or no clinical signs but may shed sufficient virus to infect naïve, unvaccinated horses, as demonstrated in an experimental study using sentinels [5,14].

It is believed that vaccine efficacy can be compromised by a mismatch between the vaccine and field virus strain(s). The antigenic evolution of EIV is closely monitored by the OIE (World Organisation for Animal Health) and associated laboratories and institutes. For this purpose, EIV sequences are analysed for antigenic variation using predictive tools and models (e.g., antigenic cartography, differentiation of viruses using haemagglutination inhibition (HI) with ferret sera). The OIE expert surveillance panel reviews the laboratory and epidemiological data on the circulation of EIV worldwide and, based on this, anticipates the impact of antigenic shift on vaccine efficacy and makes annual recommendations on the antigenic composition of EIV vaccines [4,15]. Nowadays, EIV strains belonging to the Florida Clade (FC) 1 and 2 sublineages predominate, and these have been included in the OIE recommendations for EIV vaccines since 2010, along with the recommendation that a European lineage is no longer required [15]. Since 2010, there have been no changes in the expert surveillance panel recommendations, whereas human seasonal influenza vaccines are updated annually. This is in line with previous findings that EIVs are genetically more stable than human influenza viruses [16,17].

The method of analysis of EIV antigenic evolution, on which these recommendations are based, and the criteria for updating EIV vaccines are identical to those used for human seasonal influenza vaccines [18,19]. Haemagglutination inhibition (HI) assays with ferret antisera are used, amongst other techniques, to analyse the antigenic changes in the haemagglutinin (HA) viral surface protein. Ferrets are known to be the most sensitive species for influenza virus antigenic variation and mount very strain-specific responses [18,20]. This means that ferrets can discriminate minor antigenic differences from major antigenic differences. However, these apparently large antigenic differences in EIV may not be biologically significant in horses [20,21]. This has already been observed for avian influenza where the vaccine is capable of providing sufficient protection against infection with new field strains although the vaccine virus strain differs from field virus [22,23]. It is possible that the vaccine type may play a more important role in determining EIV vaccine efficacy than minor antigenic differences between vaccine and field strains that may not be biologically relevant in the horse.

The process of updating an EIV vaccine can take years and is costly, and there is strict guidance on the requirements for demonstrating vaccine efficacy, safety, and quality that go beyond the OIE expert surveillance panel recommendations for EIV strains [24]. To date, there is only one commercially available vaccine that is able to comply with all of the current OIE expert surveillance panel recommendations. This is relatively simple for the modified vector vaccine, where the HA in the replicative defective canarypox (ALVAC) vector expressing the HA of the EIV strain in question is replaced, with HA expression being confirmed by Western blot using a monoclonal anti-EIV HA antibody, and the final vaccine construct is titrated in chicken embryo fibroblast cell culture using HA-specific immunofluorescence [25]. However, for other EIV vaccines, this is more challenging because in addition to replacing the whole EI virus or subunit, it has to be possible to differentiate the new virus strain(s) from the existing strain in the vaccine using the originally registered tests.

This study evaluated the duration of immunity at 4.5 months after the primary vaccination course (V1 and V2) of the adjuvanted, modified vector vaccine (expressing the HA gene of a FC1 and a FC2 strain) and a ISCOMatrix adjuvanted, whole inactivated virus vaccine (containing a FC1 and a European lineage strain) based on experimental infection with a recent Florida Clade 2 field strain. The aim was to provide insight into the impact of vaccine type and minor antigenic differences between vaccine and field strains on vaccine efficacy, after challenge during the most susceptible period (the immunity gap). The study showed that the two vaccines were very similar based on induced protective antibody titres (single radial haemolysis (SRH), haemagglutination inhibition (HI) and virus neutralisation (VN)) to FC1 and FC2 strains and in reducing clinical signs and viral shedding of EIV, and they provide equal protection to EIV strains currently circulating in the horse population.

## 2. Materials and Methods

### 2.1. Animals

This study was performed in 19 Norwegian Fjord horses (mares and geldings) aged between 4 and 4.5 years of age. The horses were obtained from two premises (UK and The Netherlands) and transported to a single study site (UK) where they arrived 3 weeks before EIV vaccination. Prior to transport, all horses were vaccinated against tetanus with Equilis Te (MSD Animal Health, The Netherlands) according to manufacturer’s instructions. All horses were confirmed to be seronegative for EIV based on screening of blood samples at the premises of origin and during acclimatisation at the study site. Upon arrival at the study site, horses were allocated to three rooms based on previous housing to ease mixing of horses and prevent unnecessary stress. The horses in each room were allocated randomly to a treatment group by the study investigator to ensure evenly distributed treatment groups in each of the three rooms. On day 31, the horses were moved into one room incorporating three pens to increase the space per animal, without changing the mix of treatment groups. Sample size (7 horses per vaccinated group and 5 control horses) was based on the European Pharmacopoeia criteria for EIV vaccine (inactivated), which requires at least 6 horses in the vaccinated group and at least 4 horses in the control group [26].

### 2.2. Vaccines

Two commercially available vaccines were used: (i) Equilis Prequenza (MSD Animal Health; lots A118A01 and A115A02), a whole inactivated virus vaccine containing EIV strains A/equine/Newmarket/2/93 (European lineage) and A/equine/South Africa/4/03 (FC1 sublineage), adjuvanted with ISCOMatrix (purified saponin, cholesterol and phosphatidylcholine); (ii) ProteqFlu (Boehringer Ingelheim (formerly Merial)); lots L435907 and L443492), a modified vector vaccine containing two modified recombinant canarypox vectors expressing the HA gene of EIV strains A/equine/Ohio/03 (FC1 sublineage) or A/equine/Richmond/07 (FC2 sublineage), adjuvanted with carbomer. The investigational veterinary products (IVP) were used within the stated shelf-life of the product.

### 2.3. Viruses

Virus was grown in embryonated hens’ eggs, purified, and titrated as previously described [27]. Briefly, 10–11 days old embryonated eggs were inoculated with virus and incubated for 3 days at 37 °C. Virus was harvested from the allantoic fluid, concentrated, and purified. To titrate the virus, a 10-fold serial dilution was made and used to inoculate 10–11 days old embryonated eggs. The allantoic fluid was harvested after incubation for 3 days at 37 °C and the EIV titre measured by haemagglutination assay (HA) using chicken red blood cells. The virus titre was calculated based on the number of EIV-positive and -negative eggs for each sample dilution and expressed as log_10_ EID50/mL.

EIV H3N8 A/equi-2/Wexford/14 (FC2 sublineage) was used for the experimental infection (challenge) and kindly provided by the Irish Equine Centre (Ireland). EIV H3N8 A/equine/South Africa/4/03 (FC1 sublineage) and A/equine/Meath/07 (FC2 sublineage) were used as single radial haemolysis (SRH) antigens. EIV H3N8 strains A/equine/Ohio/03 (FC1 sublineage), A/equine/South Africa/4/03 (FC1 sublineage), A/equine/Shropshire/10 (FC2 sublineage), and A/equine/Richmond/1/07 (FC2 sublineage) were used as HI and virus-neutralising (VN) antigens.

### 2.4. Vaccination and Challenge Protocol

Horses were allocated randomly into three treatment groups. Group 1 (*n* = 5) was the unvaccinated control group, group 2 (*n* = 7) received Equilis Prequenza and group 3 (*n* = 7) received ProteqFlu. Each horse from groups 2 and 3 received 1 mL (single dose) of vaccine by deep intramuscular injection (21G × 112, 0.8 × 40 mm needle) in the left neck on day 0 (V1) and in the right neck on day 28 (V2). On day 148, all horses were challenged with EIV H3N8 A/equi-2/Wexford/14 (FC2 sublineage). Each horse received 2 mL of phosphate-buffered saline-diluted allantoic fluid containing a target titre of 10^7^ egg infectious dose 50 (EID_50_) challenge virus administered intranasally by individual nebulisation using the Flexineb^®^ portable equine nebuliser (Nortev, Ireland). All these procedures were conducted at Drayton Animal Health, Stratford-upon-Avon, UK.

To ensure complete blinding, the vaccines were labelled as A and B and were not identified by their trade names. The unblinding information was only available to one individual from MSD Animal Health, who was blinded to the allocation of the animals to treatment groups. The preparation, documentation, and administration of vaccinations was performed by the study investigator who was not involved in clinical observations nor laboratory or data analysis. All of the personnel involved in clinical observations or laboratory or data analysis were blinded to the allocation of the animals to treatment groups. Unblinding took place after all of the samples and data had been analysed so that each of the treatment groups could be linked to the vaccine administered and individual animals to their treatment group.

This study was performed under the supervision of the investigator in compliance with the authorised study protocol (and amendments), standard operating procedures (SOPs), and Home Office License according to the Animal (Scientific Procedures) Act 1986.

### 2.5. Collection of Samples

Blood samples were collected from all animals on days −13/−12 (during acclimatisation), 0 (prior to vaccination), 9, 28 (prior to vaccination), 51, 86, 121, 148 (prior to challenge), 155, and 162. Blood samples were transported to the laboratory for separation of serum and stored at −15°C or below until testing. No blood sample could be collected from horse #2914166 on days 0 and 9 and horse #2904506 on day 9.

Nasal swab samples were collected from all horses on days 0 and 28 (prior to vaccination), day 147 (prior to challenge) and daily from day 149 until day 162. Swabs were taken from only one nostril per day and nostrils alternated between each sampling occasion. Transport medium (phosphate-buffered saline with foetal calf serum and antibiotics/antifungals) was added to each nasal swab and swab samples were stored at ≤−70 °C until testing. No nasal swab could be collected from horse #2910058 on days 157, 159, and 160.

### 2.6. Serology

Serum was analysed at the Irish Equine Centre (Ireland) for EIV antibodies to FC1 and FC2 using an SRH assay as previously described [28,29,30]. Serum was analysed at MSD Animal Health (The Netherlands) for EIV antibodies to FC1 and FC2 using a HI assay and for EIV neutralising antibodies using a VN assay as previously described [30,31].

For SRH, a significant rise in antibody titre was defined as an increase in the SRH of 25 mm^2^ or 50%, whichever was smaller, between paired serum samples. A poor responder was defined as a horse that did not mount a mean H3N8 SRH antibody response of >25 mm^2^ post-vaccination. HI titres were expressed as log_2_ values of the reciprocal of the highest serum dilution that gave complete inhibition of haemagglutination. A titre of >4 was regarded as significant and a two-fold increase in the titre indicative of seroconversion. A titre of <4 was converted to 3 for statistical analysis and graphical presentation. VN titres were expressed as log_2_ values of the reciprocal of the highest serum dilution that gave complete virus neutralisation. A titre of <2 was converted to 1 for statistical analysis and graphical presentation. Raw data are provided in Tables S1–S3.

### 2.7. Clinical Observations

Clinical observations, including rectal temperature, cough, nasal discharge, ocular discharge, anorexia, depression, and dyspnoea, were evaluated, recorded, and analysed using the scoring system listed in Table 1, and conducted by trained personnel who observed the horses once daily from day 146 to 162. On day 148, clinical observations were carried out prior to challenge. The horses were observed together for 20 min for cough (first coughing score), depression, and dyspnoea. After 20 min, the horses were led through a race one by one. In the race, laryngeal palpation was performed to assess cough (second coughing score), nasal and ocular discharges were assessed, and anorexia was assessed by offering concentrate feed in a bucket. A digital thermometer was used to measure rectal temperature. Rectal temperature could not be measured from horse #2907628 on day 0. Raw data are provided in Table S4A, Table S4B, Table S4C, Table S4D and Table S4E.

### 2.8. Viral Shedding

Nasal swab samples were analysed at MSD Animal Health (The Netherlands) to determine shedding of live EIV after challenge using a virus titration assay in embryonated SPF hens’ eggs [28]. Firstly, all swab samples were screened for presence of live virus and, thereafter, the amount of live virus was quantified only in the virus-positive samples.

Furthermore, nasal swab samples were analysed at the Irish Equine Centre (Ireland) to detect EIV genomic material after challenge by analysing viral RNA expression using EIV qPCR, as previously described [32]. Nasal swabs with a cycle threshold (Ct) value ≤40 were classified as positive for viral RNA.

Raw data are provided in Table S5A,B.

### 2.9. Data and Statistical Analysis

Vaccine efficacy was assessed based on the Equine Influenza Vaccine (Inactivated) 0249 Monograph [26]. According to this Monograph, the assessment of efficacy is based on vaccinated horses showing no more than mild clinical signs and control horses showing characteristic clinical signs of EIV infection. The average number of days on which the virus is shed, and the respective virus titres should be significantly lower in the vaccinated horses than in the control horses.

For the statistical analysis, descriptive statistics (i.e., arithmetic mean, plotting the data, etc.) were used to summarise trends, but no significance testing (i.e., *p*-values) was conducted. In addition, inferential statistics (i.e., regression modelling)―a statistical method where a small but representative sample is used to describe the characteristics of a larger population―was used to show whether there were significant differences between vaccinated and control animals after challenge (i.e., to evaluate vaccine efficacy to EIV challenge) using the parameters described below.

Antibody titres after vaccination were plotted, and the trends summarised using descriptive analysis. Subsequently, antibody titres after challenge were compared between groups using a linear mixed model analysis of variance (ANOVA) for repeated measures (SAS procedure *proc mixed*) using treatment group, days post-challenge (dpc), and their interaction as explanatory variables. In addition, the seroconversion trend in antibody titres after challenge were investigated for all three groups and compared between groups. The first step was fitting a linear model of the log2 antibody titre as response variable and time (i.e., dpc), treatment group, and their interaction as explanatory variables (SAS procedure *proc glm*). Subsequently, it was investigated whether it could be assumed that the slope was common to the vaccinated and control groups (i.e., the antibody titre had a similar seroconversion trend per group) or were treatment-specific (i.e., the antibody titre had a different seroconversion trend per group) by looking at whether the interaction effect was significant (treatment-specific slope) or not (common slope).

Total clinical score data (the overall of the clinical score plus the rectal temperature score) and clinical score data over time after challenge were plotted and analysed using generalised estimating equations (GEE) using a cumulative logit model, accounting for the correlation in the repeated measures of a horse (SAS procedure *proc genmod* with repeated statement). The response variable was included as an ordinal response in the GEE model using a multinomial distribution with a cumulative logic link function.

Rectal temperature data after challenge were plotted and evaluated separately from the total clinical score, and for this, the mean of the pre-challenge values was taken as baseline. Temperature data over time were evaluated using a linear mixed statistical model for repeated measures, including baseline as covariate (SAS procedure *proc mixed*). In addition, the difference in peak rectal temperature from baseline was evaluated using ANOVA.

Virus isolation data were plotted and then categorised as positive or negative and analysed as a repeated measure (pos/neg) using GEE methodology. Secondly, the viral shedding (titre in nasal swabs after challenge) was analysed by a linear mixed model ANOVA for repeated measures (SAS procedure *proc mixed*). Furthermore, the duration of virus shedding in days was counted and analysed by ANOVA. Nasal swab virus qPCR results for viral shedding was plotted and analysed by a linear mixed model ANOVA for repeated measures (SAS procedure *proc mixed*).

For all inferential statistical analyses, the level of significance α was set at 0.05 and tests were two-sided. A statistical trend approaching significance was detected when the *p*-values were between 0.05 and 0.1. Statistical software package SAS V9.4 (SAS Institute Inc., Cary, NC, USA) was used.

## 3. Results

### 3.1. Antibody Response

#### 3.1.1. SRH Antibody Response

**Figure 1:**
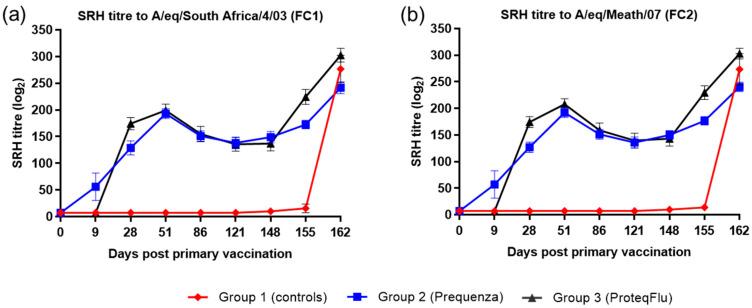
Mean single radial haemolysis (SRH) antibody titre response in groups 1–3 against H3N8 EIV (**a**) A/equine/South Africa/4/03 (FC1) and (**b**) A/equine/Meath/07 (FC2). Day 0, V1; Day 28, V2; Day 148, challenge. Red diamond = group 1, unvaccinated control group; blue square = group 2, Equilis Prequenza-vaccinated group; black triangle = group 3, ProteqFlu-vaccinated group. Error bars represent SEM.

Following V1 (day 0), horses in both vaccinated groups developed SRH titres to both EIV FC1 and FC2, which could be detected from day 9 in group 2 (Equilis Prequenza) and from day 28 in group 3 (ProteqFlu) (Figure 1). On day 28, prior to V2, SRH titres in group 3 were higher than in group 2. After V2, SRH titres were similar in the two vaccinated groups, increasing up to a peak on day 51, after which SRH titres declined in both groups until day 148 (day of challenge). After challenge, SRH titres to EIV FC1 and FC2 were higher and increased more markedly in group 3 than in group 2. SRH titres to EIV FC1 and FC2 remained negative in the control horses until after challenge when they increased markedly.

Inferential statistics showed that post-challenge seroconversion (slope of 148–162 days post-vaccination (dpv); 0–14 dpc) to EIV FC1 and FC2 did not differ significantly between groups 1 (control) and 3 but differed significantly between groups 1 and 2 (Table 2). Both vaccinated groups had significantly higher antibody titres (average of 155 and 162 dpv; 7 and 14 dpc) after challenge than the control group. After challenge, the antibody titres in group 2 were significantly lower than in group 3 (Table 3).

#### 3.1.2. HI Antibody Response

**Figure 2:**
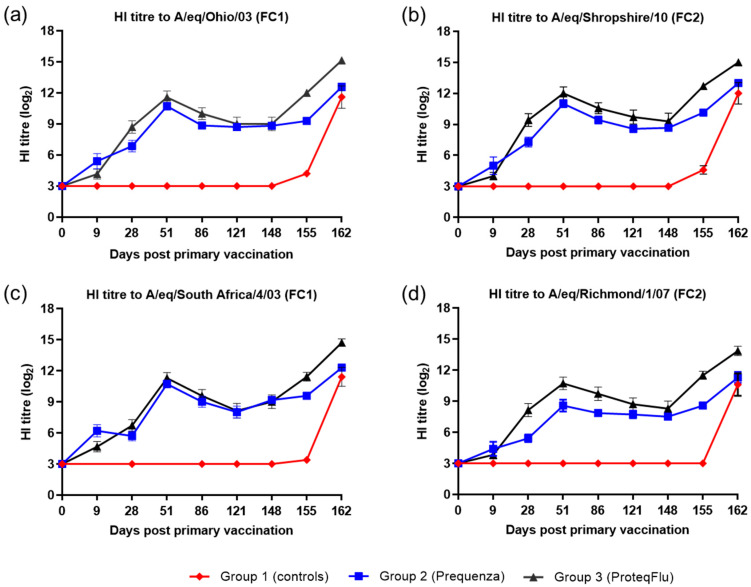
Mean haemagglutination inhibition (HI) antibody titre response in group 1–3 against H3N8 EIV (**a**) A/equine/Ohio/03 (FC1), (**b**) A/equine/Shropshire/10 (FC2), (**c**) A/equine/South Africa/4/03 (FC1) and (**d**) A/equine/Richmond/1/07 (FC2). Day 0, V1; Day 28, V2; Day 148, challenge. Red diamond = group 1, unvaccinated control group; blue square = group 2, Equilis Prequenza-vaccinated group; black triangle = group 3, ProteqFlu-vaccinated group. Error bars represent SEM. A titre of <4 was converted to 3 for graphical presentation.

On Day 0, prior to V1, all horses were seronegative, and after V1, horses in the two vaccinated groups developed measurable HI titres to both EIV FC1 and FC2 (Figure 2). On day 9, HI titres in group 2 (Equilis Prequenza) were marginally higher than in group 3 (ProteqFlu). On day 28, prior to V2, HI titres were higher in group 3 than in group 2. After V2, HI titres in both vaccinated groups increased, peaking at day 51, then declined until day 148 (day of challenge). Titres were marginally higher in group 3 than in group 2, except to A/equine/Richmond/07 (FC2) on days 28–86 when the titres in group 3 were significantly higher (very clear non-overlap of SEM) than in group 2. However, on day 148 prior to challenge, there was no difference in HI titres between the two vaccinated groups for any of the virus strains tested. After challenge, seroconversion was observed in both of the vaccinated groups. The HI titres to EIV FC1 and FC2 were higher and increased more markedly in group 3 than in group 2. HI titres to both EIV FC1 and FC2 remained negative in group 1 (controls) until after challenge when they increased markedly.

Inferential statistical analysis showed that post-challenge seroconversion (slope of days 148–162 dpv; 0–14 dpc) to EIV FC1 and to A/equine/Richmond/07 (FC2) did not differ significantly between groups 1 (control) and 3, while the slopes for A/equine/Shropshire/10 (FC2) were only significantly different between days 148 and 155 (0 and 7 dpc). The increases in HI titres (slope) to EIV FC1 and FC2 differed significantly between groups 1 and 2 for the whole post-challenge period (Table 2). After challenge, antibody titres (average of 155 and 162 dpv; 7 and 14 dpc) were significantly higher in the vaccinated groups compared to the control group. Antibody titres after challenge were significantly lower in group 2 than in group 3 (Table 3).

#### 3.1.3. VN Antibody Response

**Figure 3:**
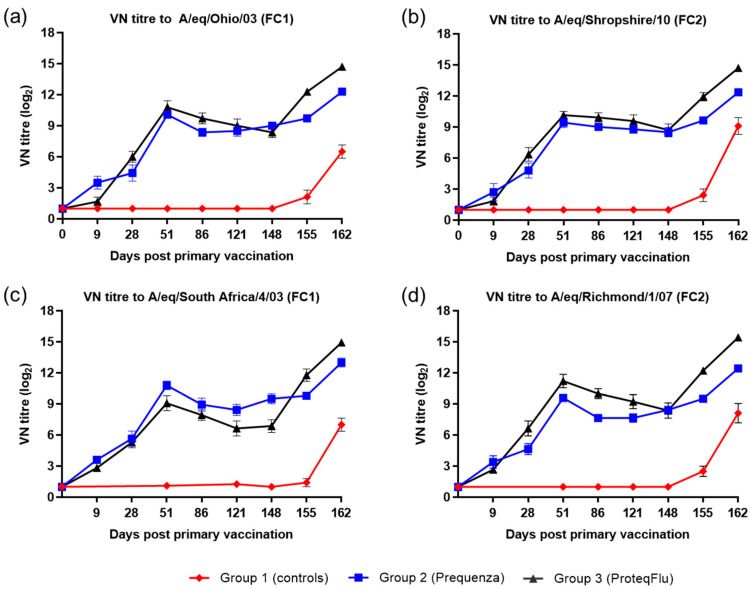
Mean virus neutralisation (VN) antibody titre response in groups 1–3 against H3N8 EIV (**a**) A/equine/Ohio/03 (FC1), (**b**) A/equine/Shropshire/10 (FC2), (**c**) A/equine/South Africa/4/03 (FC1) and (**d**) A/equine/Richmond/1/07 (FC2). Day 0, V1; Day 28, V2; Day 148, challenge. Red diamond = group 1, unvaccinated control group; blue square = group 2, Equilis Prequenza-vaccinated group; black triangle = group 3, ProteqFlu-vaccinated group. Error bars represent SEM. A titre of <2 was converted to 1 for graphical presentation.

On day 0, after V1, the horses in the vaccinated groups developed a measurable VN titre to both EIV FC1 and FC2 (Figure 3). Both vaccinated groups had a comparable VN titre profile, with a peak at day 51 followed by a decline until day 148 (day of challenge), however there were some differences in the magnitude of the VN titres. On day 9, VN titres in group 2 (Equilis Prequenza) were marginally higher than in group 3 (ProteqFlu). On day 28, prior to V2, VN titres were marginally higher in group 3 than in group 2 for all virus strains tested except A/equine/South Africa/4/03 (FC1). VN titres to A/equine/Richmond/07 (FC2) on days 28–121 and to A/equine/Ohio/03 (FC1) were higher in group 3 (ProteqFlu) than in group 2 (Equilis Prequenza) on days 28–121 and day 86, respectively. VN titres to A/equine/South Africa/4/03 (FC1) were higher in group 2 than in group 3 on days 51–148. On day 148, after challenge, seroconversion was observed in both vaccinated groups and this was more pronounced in group 3 than in group 2. VN titres to both EIV FC1 and FC2 remained negative in the control horses until after challenge, when they increased markedly.

Inferential statistical analysis showed that post-challenge seroconversion (slope of 148–162 dpv; 0–14 dpc) to EIV FC1 and FC2 did not differ significantly between groups 1 and 3, whereas the slopes for groups 1 and 2 differed significantly (Table 2). The vaccinated groups had significantly higher post-challenge antibody titres (average of 155 and 162 dpv; 7 and 14 dpc) than the control group. Antibody titres after challenge were significantly lower in group 2 than in group 3 (Table 3).

**Figure 4:**
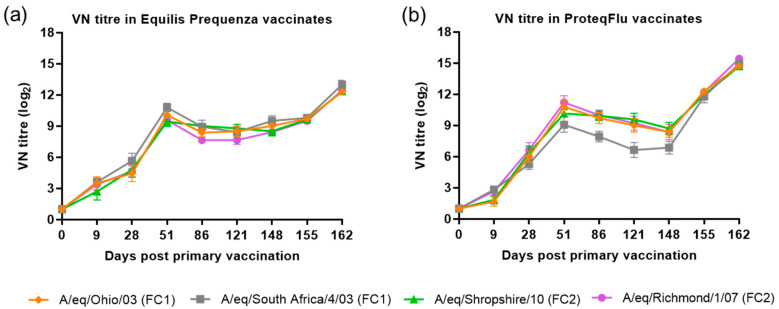
Mean virus neutralisation (VN) antibody titre response in (**a**) Equilis Prequenza-vaccinated horses and (**b**) ProteqFlu-vaccinated horses against H3N8 EIV A/equine/Ohio/03 (FC1) (red diamond), A/equine/South Africa/4/03 (FC1) (blue square), A/equine/Shropshire/10 (FC2) (green triangle) and A/equine/Richmond/1/07 (FC2) (purple dot). Day 0, V1; Day 28, V2; Day 148, challenge. Error bars represent SEM. A titre of <2 was converted to 1 for graphical presentation.

The VN titre results for the four EIV strains were compared (using descriptive statistics) between and within the two vaccinated groups (Figure 4). In group 2 (Equilis Prequenza), there were no clear differences in VN titres between the four EIV strains at any of the time points, although this vaccine contains a FC1 but not a FC2 sublineage strain. In group 3 (ProteqFlu), the VN titres to EIV strain A/eq/South Africa/4/03 (FC1) on days 51–148 were lower than the titres for the other three EIV strains. This vaccine contains the FC1 sublineage strain A/eq/Ohio/03 not the FC1 sublineage strain A/eq/South Africa/4/03 (FC1).

### 3.2. Clinical Signs of Disease after Challenge with H3N8 A/Equi-2/Wexford/14 (FC2 Sublineage)

After challenge, clinical signs of EI (nasal discharge, coughing) and increased rectal temperature were observed in all three groups.

**Figure 5:**
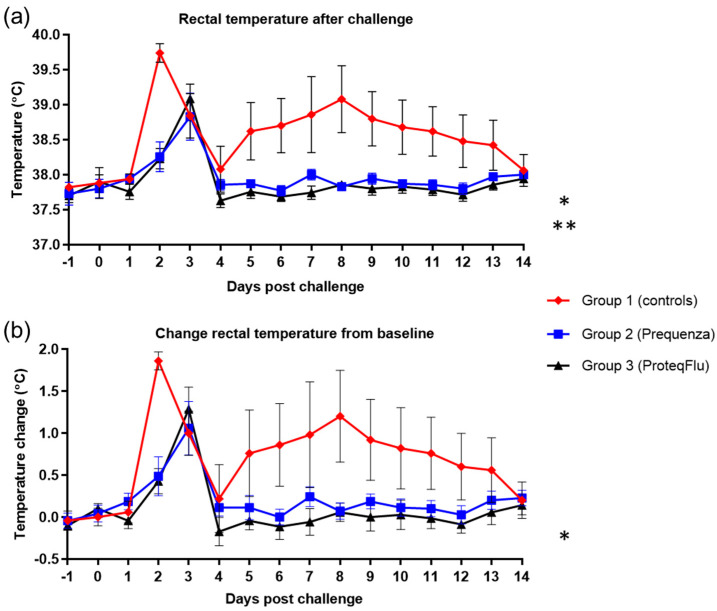
(**a**) Rectal temperature and (**b**) change of the rectal temperature from baseline in groups 1–3 after challenge with H3N8 A/equi-2/Wexford/14 (FC2 sublineage). Red diamond = group 1, unvaccinated control group; blue square = group 2, Equilis Prequenza-vaccinated group; black triangle = group 3, ProteqFlu-vaccinated group. Error bars represent SEM. Inferential statistics: significant difference (*p* < 0.05) between groups 1 and 2 (*) and groups 1 and 3 (**) (mean effect between 1 and 14 dpc).

Rectal temperature post-challenge was significantly lower (Table 4) in the two vaccinated groups (group 2, *p* < 0.0001; group 3, *p* < 0.0001) than in the control group (Figure 5) (mean effect between 1 and 14 dpc). This was also reflected in the significantly lower peak change in rectal temperature from baseline (Figure 5) in group 2 compared to group 1 (*p* = 0.0402). In group 3, the peak change in rectal temperature from baseline was also lower than in group 1, but this did not reach statistical significance, although there was a trend (*p* = 0.0547). There were no significant or notable differences in any of the rectal temperature parameters between the vaccinated groups post-challenge.

**Figure 6:**
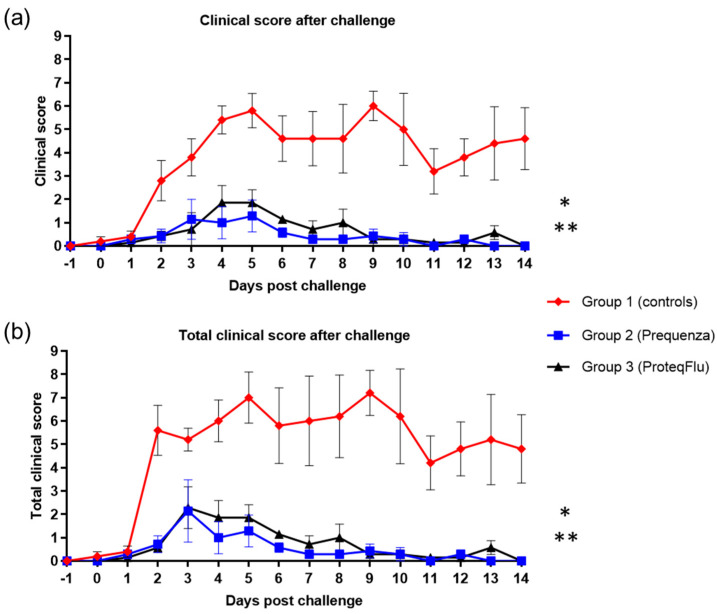
(**a**) Clinical score (clinical score excluding rectal temperature) and (**b**) Total clinical score (clinical signs and rectal temperature) in groups 1–3 after challenge with H3N8 A/equi-2/Wexford/14 (FC2 sublineage). Red diamond = group 1, unvaccinated control group; blue square = group 2, Equilis Prequenza-vaccinated group; black triangle = group 3, ProteqFlu-vaccinated group. Error bars represent SEM. Inferential statistics: significant difference (*p* < 0.05) between groups 1 and 2 (*) and groups 1 and 3 (**) (mean effect between 1 and 14 dpc).

There was a peak in clinical score on 5 and 9 dpc in the control group with average clinical scores of 5.8 and 6.0, respectively (Figure 6). In the vaccinated groups, peaks were observed on 3 and 5 dpc in group 2, with a highest average clinical score of 1.3, and on 4 and 5 dpc in group 3, with a highest average clinical score of 1.9. By 14 dpc, there were no longer clinical signs in either of the vaccinated groups, whereas the control group still had an average clinical score of 4.6. The clinical scores post-challenge were significantly lower (Table 4) in groups 2 (*p* < 0.0001) and 3 (*p* < 0.0001) than in group 1 (mean effect between 1 and 14 dpc). There were no significant differences (*p* = 0.1223) in the clinical scores between the vaccinated groups, although these tended to be lower 4–8 dpc in group 2 than in group 3.

In both vaccinated groups, the total clinical score (clinical signs and temperature) peaked at 3 dpc, and the average total clinical score was 2.3 (Figure 6). The total clinical score in the control group peaked on both 5 and 9 dpc with average total clinical scores of 7.0 and 7.2, respectively. By 14 dpc, none of the horses in either of the vaccinated groups had a total clinical score (i.e., all scores were 0), whereas the average total clinical score in the control group was 4.8. The total clinical scores post-challenge were significantly lower (Table 4) in groups 2 (*p* < 0.0001) and 3 (*p* < 0.0001) compared to the control group 1 (mean effect between 1 and 14 dpc). There was a trend (*p* = 0.0542) but not a significant difference in total clinical scores between groups 2 and 3. There was also a tendency to a lower total clinical score 4–8 dpc in group 2 compared to group 3, due to two horses in group 3 with more severe nasal discharge of longer duration.

### 3.3. Viral Shedding after Challenge with H3N8 A/Equi-2/Wexford/14 (FC2 Sublineage)

**Figure 7:**
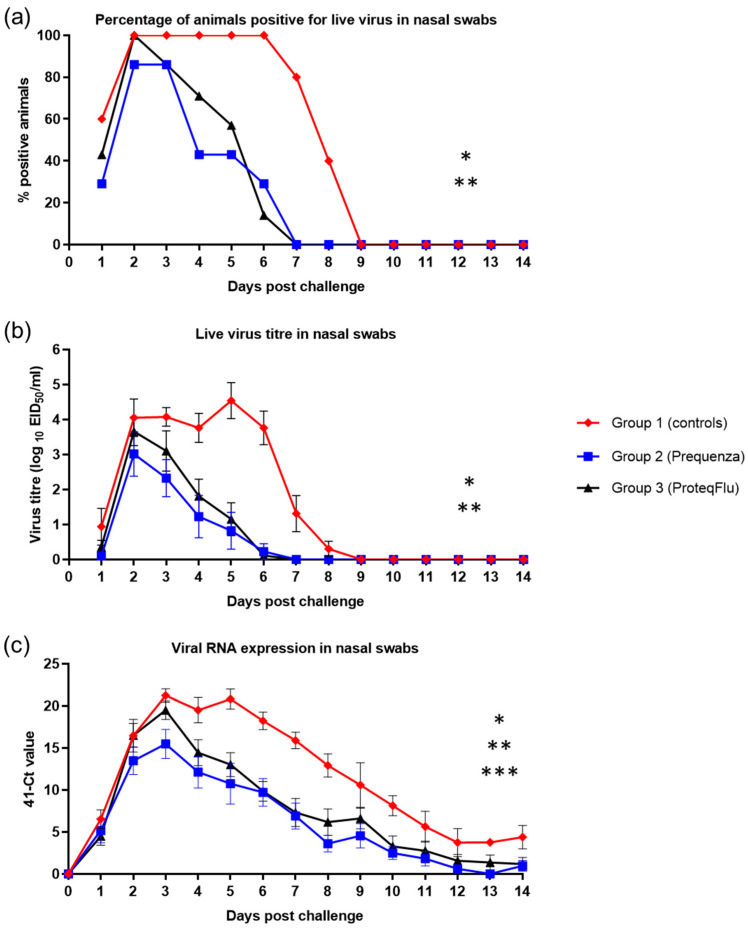
(**a**) Percentage of horses positive for live virus in nasal swabs, (**b**) Live virus titre in nasal swabs and (**c**) Amount of viral RNA expression in nasal swabs in groups 1–3 after challenge with H3N8 A/equi-2/Wexford/14 (FC2 sublineage). Red diamond = group 1, unvaccinated control group; blue square = group 2, Equilis Prequenza-vaccinated group; black triangle = group 3, ProteqFlu-vaccinated group. Error bars represent SEM. Inferential statistics: significant difference (*p* < 0.05) in the AUC between groups 1 and 2 (*), groups 1 and 3 (**) and groups 2 and 3 (***) (mean effect between 1 and 14 dpc).

Detection of live virus was analysed by percentage of horses positive for live virus and mean live virus titres. Shedding of live virus was detected from all five horses (100%) from group 1 (controls) 2–6 dpc, in all seven horses (100%) from group 3 (ProteqFlu) 2 dpc and in six out of seven horses (85.7%) from group 2 (Equilis Prequenza) 2 and 3 dpc (Figure 7). Both vaccinated groups had ceased shedding live virus by 7 dpc. All horses in group 1 had ceased shedding live virus by 9 dpc.

Titres of viable virus from nasal swabs increased in group 1 up to an average titre of 4.1 on 2 dpc and peaked at an average titre of 4.5 on 5 dpc (Figure 7). In both vaccinated groups, virus titres peaked on 2 dpc with average virus titres of 3.0 for group 2 and 3.7 for group 3. Based on virus titre, there was much lower nasal shedding of virus in the two vaccinated groups than in the control group. No viable virus was detected from all three groups from 9 dpc onwards, which is why the repeated measures ANOVA was only conducted between 1 and 8 dpc. Each of the viral shedding responses (the odds of a positive sample, the virus titre, and the duration of shedding) were significantly lower in the two vaccinated groups compared to the control group (Table 4). There was no significant difference in any of the shedding parameters between groups 2 and 3, although there was a tendency for group 2 to shed less virus than group 3, as average virus titres were lower in group 2 compared to group 3.

Genomic material of the EIV challenge strain was detected in both vaccinated groups from 1–14 dpc, peaking on 3 dpc with an average 41-cycle threshold (41-Ct) value of 15.5 for group 2 and 19.5 for group 3 (Figure 7). Only small amounts of viral RNA (41-Ct < 5) could be detected from 8 dpc in group 2 and from 10 dpc in group 3. In group 1 (control) detection of viral RNA peaked on 3 and 6 dpc with average 41-Ct values of 21.2 and 20.8, respectively, and only small amounts of viral RNA could be detected from 12 dpc. On 31 dpc, no viral RNA could be detected from group 2, while two horses in group 3 and three horses in group 1 were still positive for viral RNA. On 56 dpc, all horses were negative for viral RNA (Table S5B). There were hardly any differences detected between groups on 31 and 56 dpc, which was why the repeated measures ANOVA was only conducted between 1 and 14 dpc. The 41-Ct values in groups 2 and 3 were lower than in group 1 (*p* < 0.0001), indicating significantly less viral RNA shedding by the two vaccinated groups (Table 4). There was a significant difference between the 41-Ct values in groups 2 and 3 (*p* = 0.0367), reflecting significantly lower viral RNA shedding in group 2 than in group 3 (Table 4).

## 4. Discussion

The present study evaluated the efficacy of two commercially available EIV vaccines, an ISCOMatrix-adjuvanted, whole inactivated virus vaccine (Equilis Prequenza, group 2) or a carbomer-adjuvanted, modified vector vaccine (ProteqFlu, group 3). Serological responses, clinical signs, and viral shedding were analysed after experimental infection with a recent FC2 EI field strain at 4.5 months after the primary vaccination course (V1 and V2) and compared with unvaccinated control horses. The aim was to provide insight into the impact of vaccine type and minor antigenic differences between vaccine and field strains on vaccine efficacy, after challenge during the most susceptible period (the immunity gap). EI vaccines reduce clinical signs and viral shedding after EIV infection. In horses that have been vaccinated or infected previously, clinical signs are usually mild or inapparent. After vaccination, high antibody titres at least initially are often enough to provide protection, even where the vaccine strain differs from the current field strain. However, there have also been reports of some appropriately vaccinated horses that have developed clinical signs of influenza similar in magnitude and duration to those described for non-vaccinated horses (vaccination breakdown) following the appearance of a new field strain that differs antigenically (antigenic drift) from the EI strain(s) present in the commercially available vaccines [32,33,34,35].

It was hypothesised that due to the antigenic differences between the FC2 EI challenge strain and the virus strain(s) in the vaccine used for group 2 (Equilis Prequenza), a lower level of protection (more marked fever, more pronounced clinical signs, more marked and prolonged viral shedding, and more marked seroconversion following experimental infection) might be seen. However, following experimental infection, there were no significant differences in rectal temperature and clinical signs between the vaccinated groups and these were significantly lower than in unvaccinated controls. In addition, viral shedding was significantly reduced in both vaccinated groups compared to the unvaccinated controls. While there were differences between the two vaccinated groups in the number of horses that shed virus and the amount of viral RNA detected, there were no significant differences in viral shedding between these groups. Viable influenza virus was isolated (using embryonated hens’ eggs) from six out of seven of the horses in group 2 (Equilis Prequenza, which contains a FC1 and an European lineage strain and thus complies partly with the current OIE expert surveillance panel recommendations), and from all seven of the horses in group 3 (ProteqFlu, which contains FC1 and 2 lineage strains and thus complies fully with those recommendations). In addition, the clinical score and viral shedding pattern tended to be lower in group 2 than in group 3. There were also differences between the three groups in the excretion of EIV genomic material (PCR): in groups 1 (controls) and 3 (ProteqFlu), horses shed EIV genomic material up to 30 dpc, while in group 2 (Equilis Prequenza) horses no longer shed EIV genomic material by 14 dpc. Moreover, significantly less EIV material was shed by group 2 than by group 3. This was unexpected since group 2 was vaccinated with a vaccine that contains a FC1 and a European lineage strain and no FC2 lineage strain.

The serological response of all horses after vaccination and virus challenge was monitored since antibody titres measured by SRH and HI assays provide a correlate of protection against EI infection [4]. In addition, a VN assay, which identifies antibodies that inhibit the entry of virus into cells and virus replication, was used and has also been shown to be suitable for monitoring EIV serology after vaccination and/or infection [36,37]. These three serological assays are based on different principles but yielded comparable results, in line with previous findings [38]. Before challenge, both the serological profile and titres of the SRH and HI assay were comparable in the two vaccinated groups, irrespective of which FC sublineage strain was used in the assay. The serological profile of the VN titres in both vaccinated groups was also comparable to the SRH and HI serological profiles with two exceptions. VN titres to A/equine/Richmond/07 (FC2) on days 28–121 were lower in group 2 (Equilis Prequenza) than in group 3 (ProteqFlu) and VN titres to A/equine/South Africa/4/03 (FC1) on days 51–148 were higher in group 2 than in group 3. It has been reported previously that strain-specific antibodies give higher titres in a VN assay [39]. This likely explains the differences in VN titre that were observed, since A/equine/Richmond/07 is present in ProteqFlu (group 3) while A/equine/South Africa/4/03 is present in Equilis Prequenza (group 2).

The level of seroconversion after challenge indicates the amount of virus present, with a higher amount of virus triggering a more marked antibody response. After experimental infection, the seroconversion measured in the SRH, HI and VN assays was significantly higher in group 3 (ProteqFlu) than in group 2 (Equilis Prequenza). The increase in antibody titres (slope) after challenge was similar to the control group in group 3 but significantly lower than in the control group in group 2. Moreover, antibody titres after challenge (average of 155 and 162 dpv; 7 and 14 dpc) were significantly lower in group 2 than in group 3. This may suggest that the response to vaccination and/or immune response was likely better in group 2 than in group 3 since the same amount of virus was used to challenge each horse but there was a less pronounced serological response to challenge in group 2 than in group 3. Another unexpected finding was that VN results for group 2 were similar for all FC1 and FC2 strains, although no FC2 sublineage strain is present in the vaccine (Equilis Prequenza) used. In contrast, the vaccine used for group 3 (ProteqFlu) contains both FC1 and FC2 sublineage strains, and while similar VN results were seen for FC2 sublineage strains and FC1 A/equine/Ohio/03, this was not the case for A/equine/South Africa/4/03 (FC1). This may be due to the difference in composition of the two vaccines: one is a whole inactivated virus vaccine containing the whole virus including the conserved internal viral proteins, while the other is a vectored vaccine containing only the HA protein, which is the most immunogenic but also the most variable viral protein. A vaccine that contains only a viral subunit will be more vulnerable to antigenic changes than a whole virus vaccine. The role of antigenic changes and how these relate to vaccine efficacy and cross-reactivity will be investigated in future studies.

Humoral (utilising circulating antibodies) and cell-mediated (utilising white blood cells) immunity are the two key components of the immune system. Clinical signs of EIV infection are related to the duration and level of host chemotactic, proinflammatory and antiviral cytokines rather than virus replication. Most EIV antibodies (induced by either natural infection or vaccination) are directed towards the major viral surface structural proteins haemagglutinin (HA) and neuraminidase (NA), neutralise virus before infection occurs and inhibit the release of virus from cells after replication, thus preventing clinical signs and virus transmission. Cell-mediated immunity is also important in the control of virus infection as this is important for elimination of virus-infected cells. Cell-mediated immunity has been reported previously in EI-vaccinated horses [12,27,40]. The OIE uses a number of different antigenic analyses as a basis for its vaccine update recommendations, including the detection of changes in HA based on HI tests carried out using ferret antisera. Antisera raised in ferrets is used for this test because this species mounts a strain-specific response to influenza that picks up even minor antigenic variations. We have demonstrated that antisera raised in horses against several EIV strains cross react to a far greater extent than those raised in ferrets. In other words, the horse is able to cope with some minor antigenic variation of its own influenza virus, whereas the ferret recognises this change as significant. Our data show that even where HA from a field virus differs antigenically (antigenic drift) from that of the virus strain(s) present in a vaccine, this does not necessarily affect vaccine efficacy. Since both humoral and cell-mediated immunity play a role in protection against EIV infection, there is a need for additional methods to assess vaccine efficacy against new field strains in horses. For example, when the OIE expert surveillance panel recommends updating an EIV vaccine strain or strains, as an alternative to simply updating the strains, the existing commercially available vaccines could be evaluated against these field strains in horses to demonstrate that efficacy―including reduction of clinical signs and viral shedding―is maintained. This evaluation should be performed during the most sensitive period, the so-called immunity gap that occurs a few months after the primary vaccination course (V1 and V2).

Several different approaches to EIV vaccine development have been taken and various EIV strains have been used in different EIV vaccines over the years. Currently, three EIV vaccines are on the market in Europe: an ISCOMatrix-adjuvanted, whole inactivated virus vaccine, an ISCOM-adjuvanted inactivated virus subunit (specific viral proteins) vaccine, and a carbomer-adjuvanted modified viral vector vaccine. It is conceivable that differences in the composition of these vaccines and the mechanism in which they induce immunity could easily lead to differences in vaccine efficacy that are far more significant than the differences from simply updating the strain in a different type of vaccine. The whole inactivated virus vaccine contains all viral proteins while the modified viral vector vaccine contains only the HA gene. A viral vector vaccine triggers the immune response in a similar fashion to a single cycle of a live influenza virus infection while a whole inactivated virus vaccine needs adjuvant to trigger the immune response. However, Geeraedts et al. (2008) [41] found that the quality and quantity of the immune response triggered by a whole inactivated influenza virus vaccine and a live influenza virus was similar, suggesting that the integrity of the virus particle structure is more important for induction of an effective immune response than presence or absence of virus replication. While antibody titre is a correlate of protection against influenza virus, cell-mediated immunity also plays a role in protection, especially in cross-protection against heterologous virus strains. In mice, whole inactivated influenza virus vaccines have been shown to induce higher HI and VN titres than subunit vaccines, stimulate dendritic cells, and induce a Th1 response, providing better protection against infection [41]. Stimulation of murine dendritic cells with a non-adjuvanted, whole inactivated influenza virus vaccine in vitro resulted in a response similar to stimulation with live virus, but a non-adjuvanted, inactivated virus subunit vaccine did not [42]. In addition, whole inactivated influenza virus vaccines, but not subunit vaccines, have been shown to protect mice against lethal heterologous virus challenge through cell-mediated immunity to conserved internal influenza virus proteins [43].

Many variables play a role in the efficacy of different whole inactivated influenza virus vaccines and it is therefore important that the efficacy and safety of these vaccines be compared. For example, the method used to inactivate a whole virus can also affect vaccine efficacy due to the alterations it causes in the structure of the virus. For example, the qualitative immune response is less following inactivation with formalin than inactivation with beta-propiolactone in mice [43]. In addition, the choice of the adjuvant for a whole inactivated influenza virus vaccine is crucial because the induction of an appropriate immune response is important and differs for each adjuvant [4,44,45]. While a highly reactogenic adjuvant can lead to good vaccine efficacy, this might compromise vaccine safety and the opposite may be true for a poorly reactogenic adjuvant.

## 5. Conclusions

The difference between types of vaccine and even differences between EIV vaccines of the same vaccine type make it difficult to predict vaccine efficacy based on the antigenic difference of the HA alone, even in the face of a new field virus. Recommendations made based upon antigenic differences between a new field strain and vaccine strain(s) of EIV are a first indication that vaccine efficacy may be affected. Following such recommendations, additional testing of commercially-available vaccines against a new field strain would be required to demonstrate whether vaccine efficacy has indeed been affected, and a vaccine strain update is needed. These additional tests can be realised more rapidly than a change in vaccine composition (strain update). Changes in how to meet the recommendations for the protection of horses against EIV would increase the availability of vaccines with proven efficacy under field conditions.

## Figures and Tables

**Table 1:** The clinical observation scoring system.

Clinical Observation	Score
Temperature (°C)	0: <38.50
	1: 38.50–39.09
	2: 39.10–39.59
	3: 39.60–40.09
	4: >40.09
Nasal Discharge	0: no discharge
	1: slight serous discharge
	2: moderate discharge
	3: severe, copious discharge
	4: + if mucopurulent discharge
	4: ++ if bilateral discharge
Ocular Discharge	0: absent
	1: slight serous discharge
	2: moderate discharge
	3: severe discharge
	4: + if mucopurulent discharge
	4: ++ if bilateral discharge
Anorexia	0: eating immediately concentrates offered
	1: poorly interested in feed
	2: not interested in feed
Depression	0: active, alert, and bright
	1: depressed
	2: recumbent but still capable of getting up after stimulation
	3: paralysed or dead
Dyspnoea	0: normal respiration (<20/min)
	1: mildly increased rate (20 to <40/min)
	2: soberly increased rate (≥40/min)
Coughing	0: no coughing
	1: induced by larynx palpation (no coughing during observation period)
	2: infrequent coughing, 1–2coughing episodes during observation period
	3: severe coughing, >2 coughing episodes during observation period

**Table 2:** Results of inferential statistics: comparison of post-challenge seroconversion between groups (slope of 148–162 dpv, 0–14 dpc).

Serological Test	EIV Strain	Group Comparison (*p*-Value) 1 = Control, 2 = Equilis Prequenza, 3 = ProteqFlu	
		2 vs. 1	3 vs. 1
SRH	A/equine/South Africa/4/03 (FC1)	0.0007	0.0822
	A/equine/Meath/07 (FC2)	0.0007	0.0709
HI	A/equine/Ohio/03 (FC1)	0.0010	0.0745
	A/equine/Shropshire/10 (FC2)	0.0006	0.0211 0.1061 (148–155 dpv; 0–7 dpc)
	A/equine/South Africa/4/03 (FC1)	0.0006	0.0738
	A/equine/Richmond/1/07 (FC2)	0.0140	0.1937
VN	A/equine/Ohio/03 (FC1)	0.0113	0.8878
	A/equine/Shropshire/10 (FC2)	0.0012	0.0929
	A/equine/South Africa/4/03 (FC1)	0.0470	0.1039
	A/equine/Richmond/1/07 (FC2)	0.0071	0.9815

**Table 3:** Inferential statistics: Comparison of serological titres after challenge between groups (average of 155 and 162 dpv; 7 and 14 dpc).

Serological Test	EIV Strain	Group Comparison (*p*-Value) 1 = Control, 2 = Equilis Prequenza, 3 = ProteqFlu		
		2 vs. 1	3 vs. 1	2 vs. 3
SRH	A/equine/South Africa/4/03 (FC1)	0.0136	<0.0001	0.0025
	SRH EIV A/equine/Meath/07 (FC2)	0.0047	<0.0001	0.0010
HI	A/equine/Ohio/03 (FC1)	<0.0001	<0.0001	<0.0001
	A/equine/Shropshire/10 (FC2)	<0.0001	<0.0001	<0.0001
	A/equine/South Africa/4/03 (FC1)	<0.0001	<0.0001	0.0002
	A/equine/Richmond/1/07 (FC2)	<0.0001	<0.0001	<0.0001
VN	A/equine/Ohio/03 (FC1)	<0.0001	<0.0001	0.0001
	A/equine/Shropshire/10 (FC2)	<0.0001	<0.0001	0.0001
	A/equine/South Africa/4/03 (FC1)	<0.0001	<0.0001	0.0004
	A/equine/Richmond/1/07 (FC2)	<0.0001	<0.0001	<0.0001

**Table 4:** Inferential statistics: Comparison of the clinical observations, rectal temperature and viral shedding after challenge between groups (mean effect between 1 and 14 dpc ^a^ or 1 and 8 dpc ^b^).

Parameter	Group Comparison 1 = Control, 2 = Equilis Prequenza, 3 = ProteqFlu		
	2 vs. 1	3 vs. 1	2 vs. 3
Total Clinical Score ^a^	<0.0001	<0.0001	0.0542
Clinical Score ^a^	<0.0001	<0.0001	0.1223
Rectal Temperature over Time ^a^	<0.0001	<0.0001	0.4850
Peak Change Rectal Temperature after Challenge ^a^	0.0402	0.0547	0.8635
Viral Shedding Nasal Swabs +/− ^b^	<0.0001	<0.0001	0.2939
Viral Shedding Nasal Swab Titre ^b^	<0.0001	0.0001	0.2854
Viral Shedding Duration Days ^b^	0.0002	0.0021	0.2813
Virus *q*PCR ^a^	<0.0001	<0.0001	0.0367

^a^: effect between 1 and 14 dpc. ^b^: effect between 1 and 8 dpc. +: nasal swabs positive for EIV. −: nasal swabs negative for EIV.

## Supplementary Materials

The following are available online at https://www.mdpi.com/2076-393X/8/3/501/s1. Table S1: Single radial haemolysis results; Table S2: Haemagglutination inhibition results; Table S3: Virus neutralisation results; Table S4A: Clinical observations: Rectal temperature; Table S4B: Clinical observations: Anorexia/Depression/Dyspnoea; Table S4C: Clinical observations: Coughing A (Observation in pen)/Coughing B (Observation in race); Table S4D: Clinical observations: Nasal discharge; Table S4E: Clinical observations: Ocular discharge; Table S5A: Nasal swabs: Virus titration (live virus); Table S5B: Nasal swabs: PCR results (virus genomic material); Note 1: Horse with deviated response in group 2.

## References

[1] Landolt G., Townsend H.G., Lunn D.P., Sellon D.C., Long M.T. (2013). Equine influenza infection. Equine Infectious Diseases.

[2] The World Organisation for Animal Health (OIE) Information on Aquatic and Terrestrial Animal Diseases, Equine Influenza. https://www.oie.int/en/animal-health-in-the-world/animal-diseases/equine-influenza.

[3] Webster W.R. (2011). Overview of the 2007 Australian outbreak of equine influenza. Aust. Vet. J..

[4] Paillot R. (2014). A systematic review of recent advances in equine influenza vaccination. Vaccines.

[5] Paillot R., Prowse L., Montesso F., Stewart B., Jordon L., Newton J.R., Gilkerson J.R. (2013). Duration of equine influenza virus shedding and infectivity in immunised horses after experimental infection with EIV A/eq2/Richmond/1/07. Vet. Microbiol..

[6] Gildea S., Arkins S., Cullinane A. (2011). Management and environmental factors involved in equine influenza outbreaks in Ireland 2007–2010. Equine Vet. J..

[7] Gildea S., Arkins S., Walsh C., Cullinane A. (2011). A comparison of antibody responses to commercial equine influenza vaccines following primary vaccination of Thoroughbred weanlings—A randomised blind study. Vaccine.

[8] Cullinane A., Newton J.R. (2013). Equine influenza—A global perspective. Vet. Microbiol..

[9] Daly J.M., MacRae S., Newton J.R., Wattrang E., Elton D.M. (2011). Equine influenza: A review of an unpredictable virus. Vet. J..

[10] Wattrang E., Jessett D.M., Yates P., Fuxler L., Hannant D. (2003). Experimental infection of ponies with equine influenza A2 (H3N8) virus strains of different pathogenicity elicits varying interferon and interleukin-6 responses. Viral Immunol..

[11] Gildea S., Arkins S., Walsh C., Cullinane A. (2011). A comparison of antibody responses to commercial equine influenza vaccines following annual booster vaccination of national hunt horses—A randomised blind study. Vaccine.

[12] Paillot R., Prowse L., Montesso F., Huang C.M., Barnes H., Escala J. (2013). Whole inactivated equine influenza vaccine: Efficacy against a representative clade 2 equine influenza virus, IFNgamma synthesis and duration of humoral immunity. Vet. Microbiol..

[13] Wood J.L., Mumford J.A., Mair T.S., Slater J. (2007). Boosting in equine influenza vaccination schedules: Timing and time for a re-evaluation of requirements of national and international authorities. Vet. J..

[14] Murcia P.R., Baillie G.J., Stack J.C., Jervis C., Elton D., Mumford J.A., Daly J., Kellam P., Grenfell B.T., Holmes E.C. (2013). Evolution of equine influenza virus in vaccinated horses. J. Virol..

[15] OIE Expert Surveillance Panel on Equine Influenza Vaccine Composition, OIE Headquarters, 16 April 2020. Conclusions and Recommendations. https://www.oie.int/scientific-expertise/specific-information-and-recommendati.;ons/equine-influenza/.

[16] Hinshaw V.S., Naeve C.W., Webster R.G., Douglas A., Skehel J.J., Bryans J. (1983). Analysis of antigenic variation in equine 2 influenza A viruses. Bull. WHO.

[17] Webster R.G., Bean W.J., Gorman O.T., Chambers T.M., Kawaoka Y. (1992). Evolution and ecology of influenza A viruses. Microbiol. Rev..

[18] Cullinane A., Elton D., Mumford J. (2010). Equine influenza—Surveillance and control. Influenza Other Respir. Viruses.

[19] Mumford J., Bryant N. (2009). The Equine Influenza Expert Surveillance Panel. AHT/BEVA/DEFRA Equine Q. Dis. Surveill. Rep..

[20] Burrows R., Denyer M. (1982). Antigenic Properties of Some Equine Influenza Viruses. Arch. Virol..

[21] Horspool L.J., King A. (2013). Equine influenza vaccines in Europe: A view from the animal health industry. Equine Vet. J..

[22] Poetri O.N., Bouma A., Murtini S., Claassen I., Koch G., Soejoedono R.D., Stegeman J.A., van Boven M. (2009). An inactivated H5N2 vaccine reduces transmission of highly pathogenic H5N1 avian influenza virus among native chickens. Vaccine.

[23] Swayne D.E., Lee C.W., Spackman E. (2006). Inactivated North American and European H5N2 avian influenza virus vaccines protect chickens from Asian H5N1 high pathogenicity avian influenza virus. Avian Pathol..

[24] The European Medicines Agency (EMA) EMA/CVMP/IWP/97961/2013, Guideline on Data Requirements for Changes to the Strain Composition of Authorised Equine Influenza Vaccines in Line with Oie Recommendations. https://www.ema.europa.eu/en/documents/scientific-guideline/guideline-data-requirements-changes-strain-composition-authorised-equine-influenza-vaccines-line_en.pdf.

[25] Paillot R., Rash N.L., Garrett D., Prowse-Davis L., Montesso F., llinane A., Lemaitre L., Thibault J.C., Wittreck S., Dancer A. (2016). How to meet the last OIE expert surveillance panel recommendations on equine influenza (EI) vaccine composition: A review of the process required for the recombinant canarypox-based EI vaccine. Pathogens.

[26] (2017). Equine influenza vaccine (inactivated) monograph 0249. European Pharmacopoeia.

[27] Paillot R., Grimmett H., Elton D., Daly J.M. (2008). Protection, systemic IFNgamma, and antibody responses induced by an ISCOM-based vaccine against a recent equine influenza virus in its natural host. Vet. Res..

[28] The World Organisation for Animal Health (OIE) Equine Influenza: Chapter 3.5.7. http://www.oie.int/fileadmin/Home/eng/Health_standards/tahm/3.05.07_EQ_INF.pdf.

[29] Gildea S., Arkins S., Cullinane A. (2010). A comparative antibody study of the potential susceptibility of Thoroughbred and non-Thoroughbred horse populations in Ireland to equine influenza virus. Influenza Other Respir. Viruses.

[30] Cullinane A., Gildea S., Weldon E. (2014). Comparison of primary vaccination regimes for equine influenza: Working towards an evidence-based regime. Equine Vet. J..

[31] Pouwels H.G., Van de Zande S.M., Horspool L.J., Hoeijmakers M.J. (2014). Efficacy of a non-updated, Matrix-C-based equine influenza subunit-tetanus vaccine following Florida sublineage clade 2 challenge. Vet. Rec..

[32] Gildea S., Garvey M., Lyons P., Lyons R., Gahan J., Walsh C., Cullinane A. (2018). Multifocal Equine Influenza Outbreak with Vaccination Breakdown in Thoroughbred Racehorses. Pathogens.

[33] Martella V., Elia G., Decaro N., Di Trani L., Lorusso E., Campolo M., Desario C., Parisi A., Cavaliere N., Buonavoglia C. (2007). An outbreak of equine influenza virus in vaccinated horses in Italy is due to an H3N8 strain closely related to recent North American representatives of the Florida sub-lineage. Vet. Microbiol..

[34] Newton J.R., Daly J.M., Spencer L., Mumford J.A. (2006). Description of the outbreak of equine influenza (H3N8) in the United Kingdom in 2003, during which recently vaccinated horses in Newmarket developed respiratory disease. Vet. Rec..

[35] Barbic L., Madic J., Turk N., Daly J. (2009). Vaccine failure caused an outbreak of equine influenza in Croatia. Vet. Microbiol..

[36] Kinsley R., Scott S.D., Daly J.M. (2016). Controlling equine influenza: Traditional to next generation serological assays. Vet. Microbiol..

[37] Yamanaka T., Nemoto M., Bannai H., Tsujimura K., Matsumura T., Kokado H., Gildea S., Cullinane A. (2018). Neutralization antibody response to booster/priming immunization with new equine influenza vaccine in Japan. J. Vet. Med. Sci..

[38] Morley P.S., Hanson L.K., Bogdan J.R., Townsend H.G., Appleton J.A., Haines D.M. (1995). The relationship between single radial hemolysis, hemagglutination inhibition, and virus neutralization assays used to detect antibodies specific for equine influenza viruses. Vet. Microbiol..

[39] Haaheim L.R., Schild G.C. (1980). Antibodies to the strain-specific and cross-reactive determinants of the haemagglutinin of influenza H3N2 viruses. Antiviral activities of the antibodies in biological systems. Acta Pathol. Microbiol. Scand. B.

[40] Paillot R., Kydd J.H., Sindle T., Hannant D., Edlund Toulemonde C., Audonnet J.C., Minke J.M., Daly J.M. (2006). Antibody and IFN-gamma responses induced by a recombinant canarypox vaccine and challenge infection with equine influenza virus. Vet. Immunol. Immunopathol..

[41] Geeraedts F., Bungener L., Pool J., ter Veer W., Wilschut J., Huckriede A. (2008). Whole inactivated virus influenza vaccine is superior to subunit vaccine in inducing immune responses and secretion of proinflammatory cytokines by DCs. Influenza Other Respir. Viruses.

[42] Stoel M., Pool J., de Vries-Idema J., Zaaraoui-Boutahar F., Bijl M., Andeweg A.C., Wilschut J., Huckriede A. (2015). Innate responses induced by whole inactivated virus or subunit influenza vaccines in cultured dendritic cells correlate with immune responses in vivo. PLoS ONE.

[43] Budimir N., Huckriede A., Meijerhof T., Boon L., Gostick E., Price D.A., Wilschut J., de Haan A. (2012). Induction of heterosubtypic cross-protection against influenza by a whole inactivated virus vaccine: The role of viral membrane fusion activity. PLoS ONE.

[44] Del Giudice G., Rappuoli R., Didierlaurent A.M. (2018). Correlates of adjuvanticity: A review on adjuvants in licensed vaccines. Semin. Immunol..

[45] Olafsdottir T., Lindqvist M., Harandi A.M. (2015). Molecular signatures of vaccine adjuvants. Vaccine.

