# Supplementary material for: Determining Equine Influenza Virus Vaccine Efficacy—The Specific Contribution of Strain Versus Other Vaccine Attributes
Source: Vaccines (Basel). 2020 Sep 3;8(3):501. doi: 10.3390/vaccines8030501 (PMC7564743; doi:10.3390/vaccines8030501)
Supplement: Supplementary file 1 [file vaccines-08-00501-s001.zip › vaccines-878347-suppl.docx]

**Supplementary data EIV efficacy article.**

**Table S1.** Single radial haemolysis results.

Neg: negative, considered 7 in the graphs. *: had non-specific zones of haemolysis on the negative control. NS: no serum available for testing. SA: A/eq/South Africa/4/03 (FC1). MT: A/eq/Meath/07 (FC2)

**Table S2.** Haemagglutination inhibition results part 1.

Negative: <4, considered 3 in the table and graphs. ND: not done. NS: no serum available for testing. SA: A/eq/South Africa/4/03 (FC1). OH: A/eq/Ohio/03 (FC1). SR: A/eq/Shropshire/10 (FC2). RM: A/eq/Richmond/1/07 (FC2)

**Table S2.** Haemagglutination inhibition results part 2.

Negative: <4, considered 3 in the table and graphs. ND: not done. NS: no serum available for testing. SA: A/eq/South Africa/4/03 (FC1). OH: A/eq/Ohio/03 (FC1). SR: A/eq/Shropshire/10 (FC2). RM: A/eq/Richmond/1/07 (FC2)

**Table S3.** Virus neutralization results part 1.

Negative: <2, considered 1 in the table and graphs. 1*: samples were pooled, the pool was tested. NS: no serum available for testing. SA: A/eq/South Africa/4/03 (FC1)

OH: A/eq/Ohio/03 (FC1). SR: A/eq/Shropshire/10 (FC2). RM: A/eq/Richmond/1/07 (FC2)

**Table S3.** Virus neutralization results part 2.

Negative: <2, considered 1 in the table and graphs. 1*: samples were pooled, the pool was tested. NS: no serum available for testing. SA: A/eq/South Africa/4/03 (FC1). OH: A/eq/Ohio/03 (FC1). SR: A/eq/Shropshire/10 (FC2). RM: A/eq/Richmond/1/07 (FC2)

**Table S4A.** Clinical observations: Rectal temperature.

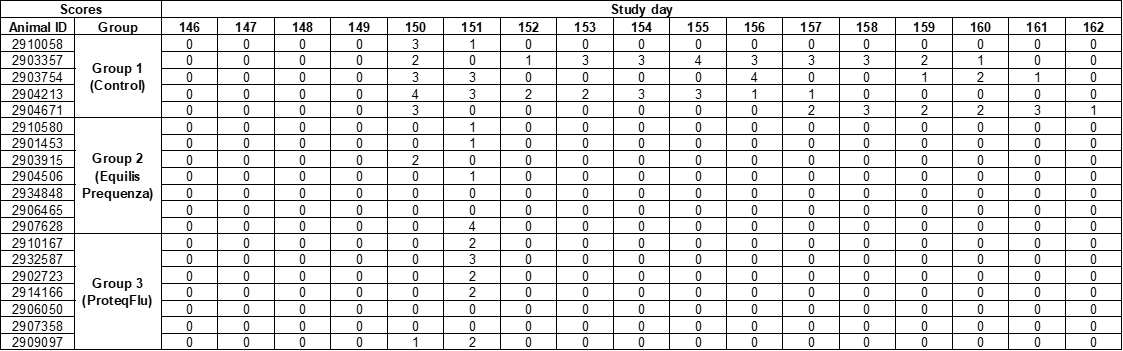


Temperature Key: 0: < 38.50; 1 - 38.50 – 39.09°C; 2 - 39.10 – 39.59°C; 3 - 39.60 – 40.09°C; 4 >40.09°C

**Table S4B.** Clinical observations: Anorexia/Depression/Dyspnoea.

Anorexia: 0 – Animal immediately eating the concentrates offered, 1 – Poorly interested in feed, 2 – Not interested in feed

Depression: 0 – Active, alert and bright, 1 – Depressed animals, 2 – Recumbent but still capable of getting to their feet after stimulation, 3 – Paralysed or dead animal

Dyspnoea: 0 – Normal respiration (<20/min), 1 – Mildly increased rate (20 to <40/min), 2 – Severely increased (≥40/min)

**Table S4C.** Clinical observations: Coughing A (Observation in pen) / Coughing B (Observation in race).

Coughing A: 0 – No coughing, 1 - Induced by larynx palpation (no other coughing during observation period), 2 – Infrequent cough, 1-2 coughing episodes during the observation period, 3 – Severe cough, more than 2 coughing episodes during the observation period

Coughing B: 0 – No coughing, 1 - Induced by larynx palpation (no other coughing during observation period), 2 – Infrequent cough, 1-2 coughing episodes during the observation period, 3 – Severe cough, more than 2 coughing episodes during the observation period

**Table S4D.** Clinical observations: Nasal discharge.

Key: 0 – No discharge; 1 – slight serous discharge; 2 – Moderate discharge; 3 – Severe, copious discharge; +1 if mucopurulent discharge; +2 if bilateral discharge

Key: 0 – No discharge; 1 – slight serous discharge; 2 – Moderate discharge; 3 – Severe, copious discharge; 4 - +1 if mucopurulent discharge; 4 - +2 if bilateral discharge

**Table S4E.** Clinical observations: Ocular discharge.

Key: 0 – No discharge; 1 – slight serous discharge; 2 – Moderate discharge; 3 – Severe, copious discharge; +1 if mucopurulent discharge; +2 if bilateral discharge

**Table S5A.** Nasal swabs: Virus titration (live virus).

0= negative; 1= positive; NS = No sample taken

NS = No sample taken.

**Table S5B.** Nasal swabs: PCR results (virus genomic material).

* = Where PCR results are negative no CT value is available; NS = No sample taken.

Note 1. Horse with deviated response in group 2

Horse 2907628 (group 2) was fractious and difficult to vaccinate and take samples from (blood samples could not be taken at several time points). The antibody titres in this horse were much lower than the other horses in the same group and this was also the only horse in this group that had a rectal temperature >40°C after challenge. Although, one could speculate that this horse had not been vaccinated properly, the data from this horse have been included in the analysis. The clinical score is significantly lower in group 2 than in group 3 when this horse excluded from the analysis.
